# The Use of Silver Solid Amalgam Electrodes for Voltammetric and Amperometric Determination of Nitrated Polyaromatic Compounds Used as Markers of Incomplete Combustion

**DOI:** 10.1100/2012/231986

**Published:** 2012-04-30

**Authors:** Oksana Yosypchuk, Jindřich Karásek, Vlastimil Vyskočil, Jiří Barek, Karolina Pecková

**Affiliations:** UNESCO Laboratory of Environmental Electrochemistry, Department of Analytical Chemistry, Faculty of Science, Charles University in Prague, Albertov 6, 128 43 Prague 2, Czech Republic

## Abstract

Genotoxic nitrated polycyclic aromatic hydrocarbons (NPAHs) are formed during incomplete combustion processes by reaction of polycyclic aromatic hydrocarbons (PAHs) with atmospheric nitrogen oxides. 1-Nitropyrene, 2-nitrofluorene, and 3-nitrofluoranthene as the dominating substances are used as markers of NPAHs formation by these processes. In the presented study, voltammetric properties and quantification of these compounds and of 5-nitroquinoline (as a representative of environmentally important genotoxic heterocyclic compounds) have been investigated using a mercury meniscus modified silver solid amalgam electrode (m-AgSAE), which represent a nontoxic alternative to traditional mercury electrodes. Linear calibration curves over three orders of magnitude and limits of determination mostly in the 10^−7^ mol L^−1^ concentration range were obtained using direct current and differential pulse voltammetry. Further, satisfactory HPLC separation of studied analytes in fifteen minutes was achieved using 0.01 mol L^−1^ phosphate buffer, pH 7.0 : methanol (15 : 85, v/v) mobile phase, and C_18_ reversed stationary phase. Limits of detection of around 1 *·* 10^−5^ mol L^−1^ were achieved using amperometric detection at m-AgSAE in wall-jet arrangement for all studied analytes. Practical applicability of this technique was demonstrated on the determination of 1-nitropyrene, 2-nitrofluorene, 3-nitrofluoranthene, and 5-nitroquinoline in drinking water after their preliminary separation and preconcentration using solid phase extraction with the limits of detection around 1 *·* 10^−6^ mol L^−1^.

## 1. Introduction

Materials based on solid or paste amalgams are of increasing importance in electroanalysis of reducible analytes. Solid amalgam electrodes (SAE) were reintroduced in electrochemistry in 2000 [1, 2] and represent electrochemically the most similar alternative to mercury electrodes due to comparable cathodic potential window and relatively high sensitivity. The additional benefits of SAE include nontoxicity of amalgam materials and mechanical robustness that allows their application in liquid flow systems. Among all the metals forming amalgams, silver seems to perform best for analytes missing specific interactions (e.g., complexation with metal cations) with metals of the amalgam [3, 4]. Silver solid amalgam electrodes (AgSAE) modified by mercury meniscus (m-AgSAE) were shown to be the most convenient alternative to mercury electrodes regarding sensitivity, limits of determination, and repeatability for a number of reducible organic analytes (see reviews [5–8]) including nitro derivatives of aromatic compounds [5–7, 9]. Beside numerous voltammetric studies, the applicability of AgSAE for detection in liquid flow methods was demonstrated previously in flow injection analysis (FIA) for 5-nitroquinoline (5-NQ) [10] and N-nitroso antineoplastic drugs [11] or in HPLC after separation of selected nitrophenols [12] using wall-jet or thin-layer arrangement of the detection cell.

In the presented study, the analytical applicability of m-AgSAE is demonstrated in batch voltammetry and flow-through amperometry of selected nitro derivatives of polycyclic aromatic hydrocarbons (NPAHs) used as markers of incomplete combustion processes [13]—1-nitropyrene (1-NP), 2-nitrofluorene (2-NF), and 3-nitrofluoranthene (3-NFt)—and of 5-NQ as a representative of polycyclic aromatic nitrogen-containing heterocycles, recognized as a relatively new class of environmental pollutants with adverse health effect [14] formed by incomplete combustion [15]. All studied compounds are formed by electrophilic nitration reactions of parent PAHs in the presence of NO_2_ during combustion [16, 17]. Because of their volatility and polarity, formed primary NPAHs are distributed between the vapor and particulate phase of combustion gases, where the studied compounds 1-NP, 2-NF, and 3-NFt are usually the dominating substances [13]. These derivatives differ from secondary NPAHs formed in the atmosphere by the radical-initiated gas-phase reactions of parent PAHs, that is, during daylight hours the attack of OH radicals to gaseous PAHs, followed by addition of NO_2_ and by water molecule elimination, and during nighttime hours the attack of NO_2_ to the PAH, followed by reaction with NO_2_ and loss of nitric acid. Typically, 2-nitrofluoranthene, 2-nitropyrene, and 3-nitrofluorene are formed by these processes [13, 18–21].

Both formation ways contribute to ubiquitous presence of NPAHs in the environment. Their occurrence was also reported in photocopier toners, cigarette smoke, and certain food items, such as grilled meats, teas, and coffees [13, 22, 23].

The most frequently methods used for NPAHs determination were subject of several reviews [13, 17, 23, 24] and include (i) mass spectrometric techniques combined with gas chromatography, liquid chromatography, or electron capture mode; (ii) gas chromatography combined with the flame ionization or the thermionic detector, electron capture detector, chemiluminiscence-based thermal energy analyzer, or nitrogen-phosphorus selective detector; (iii) HPLC combined with fluorescence (FD), chemiluminiscence, or electrochemical detection (ED). The FD is applicable only after reduction of NPAHs to amino derivatives, because NPAHs produce very low yield of fluorescence after irradiation by the UV light, as a result of the strong electron withdrawing effect of a nitro group. A popular approach relies on the electrochemical online or offline reduction of NPAHs prior FD [25–28].  Table 1 offers overview of representative electrochemical and liquid flow methods for determination of studied NPAHs in standard solutions. The lowest of reported limits of detection (*L*_*D*_) for selected HPLC methods are able to compete with the *L*_*D*_s obtained by GC/MS in negative ion chemical ionization (NICI) and selective ion monitoring (SIM) mode, the preferred NPAH analysis methods in recent years [17]. The drawback of liquid chromatographic techniques, that is, the lower resolution is usually compensated by easier handling of samples compared to GC methods, as these often require time-consuming sample-purification or derivatization procedures. This is of great importance when considering that the environmental samples are usually very complex and the concentrations of NPAHs differ substantially from sample to sample, thus the analytical methods must include an extensive sample cleanup and preliminary extraction, prefractionation, and preconcentration step.

In the presented study, the analytical applicability of m-AgSAE is demonstrated in batch voltammetric analysis of 1-NP, 2-NF, 3-NFt, and 5-NQ. Their electrochemical behaviour was characterized using cyclic voltammetry (CV) and compared to that using mercury electrodes. Furthermore, a differential pulse voltammetric (DPV) and a direct current voltammetric (DCV) method were developed for their sensitive determination at m-AgSAE. Moreover, the separation of tested analytes using HPLC on reversed C_18_ phase and possibility of the combination of HPLC with amperometric detection at m-AgSAE in wall-jet arrangement is presented. Applicability of this technique was demonstrated on the determination of 1-NP, 2-NF, 3-NFt, and 5-NQ in model drinking water samples after their preliminary separation and preconcentration using solid phase extraction (SPE).

## 2. Experimental

### 2.1. Reagents

The stock solution of 1-NP (purity 99%), 2-NF (98%), and 3-NFt (99%) were prepared by dissolving of the pure substance in 100 mL of methanol, the stock solution of 5-NQ (99%) in 100 mL of deionized water. The concentration of all stock solutions was 1 · 10^−3^ mol L^−1^, the pure substances were supplied by Sigma-Aldrich, Prague, CZ. All stock solutions were stored in glass vessels in the dark. It followed from a spectrophotometric study of the stability of all stock solutions that they are stable for at least 90 days.

Britton-Robinson (BR) buffers were prepared in a usual way, that is, by mixing a solution of 0.04 mol L^−1^ in phosphoric acid, 0.04 mol L^−1^ in acetic acid, and 0.04 mol L^−1^ in boric acid with the appropriate amount of 0.2 mol L^−1^ sodium hydroxide solution (all chemicals Lachema, Brno, CZ). Methanol (Merck, Prague, CZ) of gradient grade purity was used for mobile phase preparation. Deionized water was produced by Milli-Q_plus_ system (Millipore, Billerica, MA, USA).

### 2.2. Apparatus

Voltammetric measurements were carried out using computer-controlled Eco-Tribo-Polarograph with Polar Pro software, version 5.1 for Windows 95/98/Me/2000/XP (both Eco-Trend Plus, Prague, CZ) in combination with a three-electrode arrangement with a platinum wire auxiliary electrode and silver∣silver chloride (1 mol L^−1^ KCl) reference electrode (both Monokrystaly, Turnov, CZ), to which all the potential values are referred. As the working electrode, m-AgSAEs with the disc diameter of 0.55 mm (geometric area 0.237 mm^2^) for 5-NQ or 0.50 mm (geometric area 0.196 mm^2^) for 1-NP, 2-NF, and 3-NFt were used. The m-AgSAE consisted of a drawn-out glass tube, the bore of which near the tip was filled with a fine silver powder, amalgamated by liquid mercury and connected to an electric contact [3]. Afterwards, it was immersed into a small volume of liquid mercury and agitated for 15 s. The m-AgSAE could be used for several weeks without major changes, only its regeneration through mechanical contact with mercury was recommended to be repeated every week.

The HPLC system consisted of a high-pressure pump L-2130 HTA and a diode-array detector (DAD, L-2450, all Merck-Hitachi, Whitehouse Station, NJ, USA) controlled by EZChrom Elite (Agilent Technologies, Santa Clara, CA, USA) software working in Windows XP (Microsoft Corporation, Redmond, WA, USA). The column KROMASIL (250 × 4.6 mm, 7 *μ*m) with reversed C_18_ phase (Phenomenex, Torrance, CA, USA) was used. Samples degassed for 3 min by passing nitrogen (purity 4.0, Linde, Prague, CZ) were injected manually using 20 *μ*L Rheodyne (IDEX Health & Science, Rohnert Park, CA, USA) injection valve. The mobile phase was degassed by ultrasonication using PS 02000A ultrasonic bath (Notus-Powersonic, Vráble, Slovakia) followed by passing nitrogen (purity 4.0, Linde, Prague, CZ) continuously for the whole measurement period. The measurements were carried out at laboratory temperature (22°C). Electrochemical detector in “wall-jet” arrangement described earlier [5] with three-electrode system was used. The working electrode was m-AgSAE with the disc diameter of 0.55 mm. Ag∣AgCl (1 mol L^−1^ KCl) reference and platinum wire auxiliary electrodes (both Monokrystaly, Turnov, CZ) were used. The electrode surface-capillary outlet distance was kept at 0.5 mm. The electrode system was driven by ADLC 1 (Laboratorní přístroje, Prague, CZ) potentiostat. For the spectrophotometric detection, the wavelength *λ* = 254 nm was used.

The pH was measured by pH meter Jenway 4330 with combined glass electrode (type 924005; Jenway, Chelmsford, UK). The pH meter was calibrated with standard pH buffers (Sevac, Prague, CZ). The pH values refer to those of the aqueous phase, the pH^f^ values refer to those of the resulting pH of the mixtures of the aqueous phase with the organic solvent.

### 2.3. Procedures

During voltammetric measurements, the following m-AgSAE pretreatment was used: before starting the work, as well as after every pause longer than one hour, the electrochemical activation of m-AgSAE was carried out in stirred 0.2 mol L^−1^ KCl at –2200 mV for 300 s; afterwards, the electrode was rinsed with deionized water. Further, regeneration of m-AgSAE lasting about 30 s preceded each scan; this included the application of 300 polarizing cycles, representing the switching of the working potential from *E*_in_ to *E*_fin_ for 50 ms. *E*_in_ was selected about 50–100 mV more negative than the potential of the anodic dissolution of the electrode material, *E*_fin_ was selected about 50–100 mV more positive than the potential of the hydrogen evolution in the given supporting electrolyte. Under these conditions, eventual oxides of mercury or silver are reduced and adsorbed molecules are desorbed. For CV, the regeneration procedure was applied before the first cycle. The appropriate values of the potential and the time of regeneration were inset and modified in the program of the used computer-controlled instrument and regeneration of m-AgSAE could thus be carried out automatically. For DPV measurements, the pulse amplitude of –50 mV, the pulse duration of 100 ms, the sampling time of 20 ms beginning 80 ms after the onset of the pulse, and the interval between pulses of 100 ms were applied. The scan rates of 20 mV s^−1^ in DC and DP voltammetric experiments and 100 mV s^−1^ in CV were used.

The general procedure to obtain voltammograms was as follows: a required amount of the stock solution of the investigated substance was placed in a 10 mL volumetric flask, an appropriate volume of methanol was added, and the system was diluted to volume with a BR buffer of the required pH. Oxygen was removed from the measured solutions by purging with nitrogen for five minutes.

In the case of HPLC-ED, the mixture of 0.01 mol L^−1^ phosphate buffer, pH 7.0: methanol (15 : 85, v/v) was used as mobile phase. Diluted analyte solutions were prepared by diluting of exact volume of the stock solutions with mobile phase. The flow rate *F*_*m*_ was set at 1 mL min^−1^ and the injected sample volume *V*_inj_ was 20 *μ*L. The m-AgSAE in HPLC experiments was activated similarly as in voltammetric measurements in 0.2 mol L^−1^ KCl once a week or when new mercury meniscus was formed. No other electrochemical pretreatment was performed prior to each injection.

The determination of 1-NP, 2-NF, 3-NFt, and 5-NQ in model drinking water samples after SPE using LiChrolut RP-18 E 500 mg/3 mL (Merck, Darmstadt, Germany) cartridges was performed as follows: the SPE column was activated using vacuum manifold by washing with 3 mL of methanol. Afterwards, 100 mL of the sample was sucked through the column. This step was followed by air sucking through the column for 1 min for drying and elution with 1 mL of methanol. The eluent was always allowed to pass through the columns without the use of vacuum. 20 *μ*L of the eluate were directly injected into the HPLC system. Drinking water from public water pipeline in the building of Faculty of Science of Charles University in Prague spiked with appropriate amount of 1-NP, 2-NF, 3-NFt, and 5-NQ stock solution was used as a model sample. The recoveries were calculated for four samples (*V* = 100 mL, *c* = 1 · 10^−6^ mol L^−1^) from the ratio *I*_*p*_/*I*_*p*_^*o*^ , where *I*_*p*_ is the height of the peak of the analyte of interest after solid phase extraction and *I*_*p*_^*o*^ is the height of peak in a reference solution prepared by the addition of the standard solution of studied analyte to the blank solution.

The parameters of calibration curves (e.g., slope, intercept, and correlation coefficient) were calculated using statistic software Origin Pro 6.0 (OriginLab Corporation, Northampton, MA, USA). In voltammetric experiments, the limit of quantification (*L*_*Q*_) was calculated using the standard deviation of the mean of the peak heights obtained for seven consecutive determinations of lowest measurable concentration (*s*_*c*_) and the slope *b* of the analytical curve related by: *L*_*Q*_ = 10*s*_*c*_/*b* [38]. In HPLC, the limit of detection (*L*_*D*_) was calculated from the peaks heights as the concentration of an analyte which gave a signal three-times the background noise (*S*/*N* = 3). All the statistical data are calculated for the level of significance *α* = 0.05.

## 3. Results and Discussion

The electrochemical reduction of studied analytes is influenced by the presence of an organic solvent in the supporting electrolyte, which is necessary because the tested analytes (except 5-NQ) are sparingly soluble in water. From the analytical point of view, the minimum amount of organic solvent in the analyzed solutions is desirable with respect to the safety and economic issues in analytical praxis. Therefore, prior all the electrochemical experiments the solubility of the tested compounds at *c* = 1 · 10^−4^ mol L^−1^ in mixed aqueous-methanolic media was investigated. The pH of the aqueous phase does not play any key role due to the aprotic character of the studied analytes. The ratios of aqueous phase-methanol necessary for dissolution were 1 : 1, 3 : 7, and 1 : 9 for 2-NF, 1-NP, and 3-NFt, respectively, that is, the more extended aromatic system requires higher organic phase content as expectable. These ratios were kept during all the electrochemical experiments.

The results for CV, and DC and DP voltammetric determination of 5-NQ in aqueous media at m-AgSAE were published recently [10] and the original source is referred throughout the following section.

### 3.1. Cyclic Voltammetry

Firstly, cyclic voltammograms (CVs) of 1-NP, 2-NF, and 3-NFt in acidic, neutral, and alkaline media were recorded to characterize their redox reactions at m-AgSAE. As supporting electrolyte, the mixture of BR buffer (pH 2.2 and 7.0) or 0.01 mol L^−1^ NaOH (pH 12.0) with methanol was used. CVs of 5-NQ in aqueous media were described previously [10] and are consistent with the CVs described in this section.

**Figure 1:**
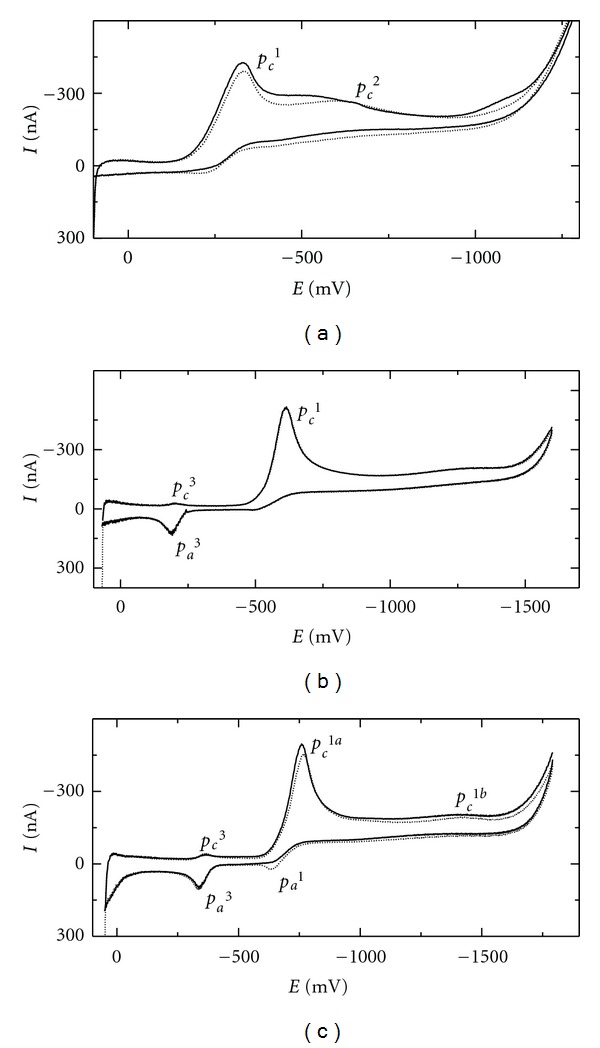
Cyclic voltammograms of 1-NP (*c* = 1 · 10^−4^ mol L^−1^) at m-AgSAE in methanol—BR buffer or 0.01 mol L^−1^ NaOH mixture (7 : 3). pH of BR buffer (a) 2.0 (pH^*f*^ 2.3); (b) 7.0 (pH^*f*^ 8.5); (c) pH of 0.01 mol L^−1^ NaOH 12.0 (pH^*f*^ 12.1). The 1st (—), and the 12th (⋯) scan, scan rate 100 mV s^−1^.

In Figure 1, the first and the twelfth cycles are presented for 1-NP, the CVs of 2-NF and 3-NFt featured similar course. In acidic (Figure 1(a)) and neutral medium (Figure 1(b)), the mechanism is presumably analogous to that earlier recognized for nitrobenzene and NPAHs at mercury electrodes [39, 40] and for 1- and 2-nitronaphthalene at m-AgSAE [41]. It includes the main four-electron reduction of the nitro group (ArNO_2_) to the hydroxylamino group ArNHOH; cathodic peak *p*_*c*_^1^:

(1) $${\text{ArNO}}_{2}+4e^{-}+4{\text{H}}^{+}\to \text{ArNHOH}+{\text{H}}_{2}\text{O}$$

The peak pair *p*_*c*_^3^/*p*_*a*_^3^ at more positive potentials than *p*_*c*_^1^ corresponds to the two-electron reversible oxidation of the formed hydroxylamino group to the nitroso one in the anodic scan (2) and its consecutive reduction in the following cathodic scan. The peak potential difference (Δ*E* = *E*_*pc*3_ − *E*_*pa*3_) of about 30 mV for neutral and alkaline media for all tested compounds confirms the tendency to reversibility of this process, although the peak heights ratio *p*_*c*_^3^/*p*_*a*_^3^ is smaller than one. In acidic media, this peak pair is hardly observable due to the limited potential window in the anodic region.

(2) $$\text{ArNHOH}⟷\text{ArNO}+2e^{-}+2{\text{H}}^{+}$$

The presence of a second cathodic peak *p*_*c*_^2^ in acidic media indicates presumably the two-electron reduction of protonated ArNHOH to corresponding amine ArNH_2_ (3) as we proved for the reduction of 1-nitronaphthalene at m-AgSAE [41]:

(3) $$\text{ArNHOH}+2e^{-}+2{\text{H}}^{+}\to {\text{ArNH}}_{2}+{\text{H}}_{2}\text{O}$$

In alkaline aqueous or mixed aqueous-organic media, the situation is rather more complicated and more peaks usually appear at recorded CVs. For nitrobenzene reduction at mercury electrodes, several articles outlined the possibility to form electroactive azoxybenzene by the reaction of nitrosobenzene with phenylhydroxylamine which can be acid and/or base catalyzed [42–44]. The reduction of possibly formed azoxybenzene can lead to hydrazobenzene which can be oxidized to azobenzene. These and other electroactive species may be formed and give the voltammetric signal [39]. Similar behavior was described for 3-NFt at HMDE [45]. Moreover, this analyte features in mixed methanol-alkaline media another cathodic peak at about −1600 mV (versus Ag∣AgCl, 1 mol L^−1^ KCl). It was shown by potentiostatic coulometry that this peak corresponds to the reduction of ArNHOH to ArNH_2_ (3), otherwise typical for acidic media [34].

The other reduction mechanism was recognized in the presence of surfactants and in aprotic solvents at mercury electrodes, or in mixed organic-aqueous alkaline media at solid electrodes [39, 44] including m-AgSAE [41]. A fast one-electron uptake by nitro group to form nitro radical anion ArNO_2_^∙^^−^ (4) is followed by irreversible three-electron reduction of ArNO_2_^∙^^−^ to hydroxylamino group (5):

(4) $$\begin{array}{c} {\text{ArNO}}_{2}+e^{-}⟷{\text{ArN}{\text{O}}_{2}^{∙}}^{-} \end{array}$$

(5) $$\begin{array}{c} {\text{ArN}{\text{O}}_{2}^{∙}}^{-}+{3e}^{-}+4{\text{H}}^{+}\to \text{ArNHOH}+{\text{H}}_{2}\text{O} \end{array}$$

This mechanism relays on the stabilization of ArNO_2_^∙^^−^ in the media with the lack of protons together with inhibited electron transfer. In the case of ArNO_2_ reduction in mixed aqueous-organic alkaline media at m-AgSAE, the inhibition effect was ascribed to the organic solvent (methanol) adsorbing at the solid surface of amalgam electrode [41].

**Figure 2:**
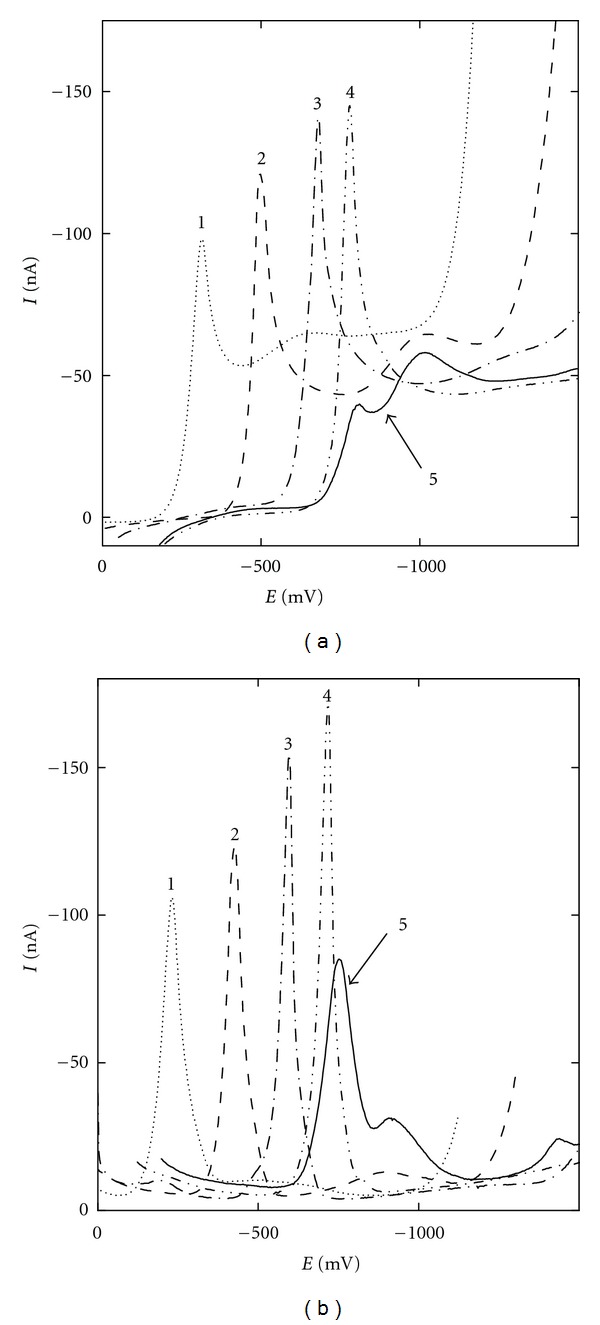
Selected DC voltammograms (a) and DP voltammograms (b) of 2-NF (*c* = 1 · 10^−4^ mol L^−1^) at m-AgSAE in a methanol-aqueous phase mixture (1 : 1). pH of the aqueous phase: 2.0 (1), 5.0 (2), 8.0 (3), 11.0 (4) for BR buffer, and 13.0 (5) for 0.1 mol L^−1^ NaOH.

For 1-NP, this splitting of the nitro group reduction into two separated processes is only insinuated in alkaline media (Figure 1(c)) by the main cathodic peak *p*_*c*_^1*a*^ (4) and indistinctive peak *p*_*c*_^1*b*^ corresponding to (5). Also the presence of the anodic peak *p*_*a*_^1*a*^ presumably corresponding to the oxidation of ArNO_2_^∙^^−^ to ArNO_2_ accordingly to (4) reveals that the inhibition process leading to the peak splitting is partially present. Similarly, CVs of 2-NF and 3-NFt exhibit only insinuated peaks *p*_*c*_^1*b*^. Clear separation of the one-electron (4) and three-electron reduction process (5) is obvious at DC voltammograms of 2-NF at pH values higher than 11.0 in Figure 2(a). The ratio 1 : 3 was estimated for the area under the DC voltammetric peaks recorded in the most alkaline media with aqueous phase of pH 13.0. Both processes are pH independent: while the first step (4) is proton-free, in the second one (5), the protonization follows the rate determining step—the uptake of the first electron. At pH values lower than 11.0, the reduction peak potential shift toward negative values with increasing pH (see Figure 2), as the rate determining step, presumably the uptake of the second electron in (1) as supposed for mercury electrodes [40], is preceded by the protonization in the above described four-electron process.

 More extended study of mechanism of the redox processes at m-AgSAE would be necessary to confirm presented assumptions and some other features, for example, the presence of the third cathodic peak of 3-NFt in alkaline media, placed at the onset of supporting electrolyte decomposition, similarly as at mercury electrodes [34].

### 3.2. DC Voltammetry and Differential Pulse Voltammetry

The development of a DC and DP voltammetric method for the determination of 1-NP, 2-NF, and 3-NFt included optimization of the pH and composition of the supporting electrolyte and further characterization of the analytical parameters (repeatability, linear dynamic range, and *L*_*Q*_). The aqueous : organic phase (methanol) ratio in supporting electrolyte was again 1 : 1, 3 : 7, and 1 : 9 for 2-NF, 1-NP, and 3-NFt, respectively. 5-NQ was determined in 0.05 mol L^−1^ borate buffer pH 9.0 [10].

The influence of pH on DC and DP voltammograms of 2-NF in BR buffer, pH 2.0–12.0, and pH 13.0 in 0.1 mol L^−1^ NaOH is depicted in Figures 2(a) and 2(b), respectively. For 3-NFt (voltammograms not shown), the situation is similar. The curves exhibit two peaks from acidic to neutral media (pH 2.0–7.0 for 2-NF, pH 2.0–4.0 for 3-NFt), and strong alkaline media (pH 13.0 for 2-NF, pH 10.0–12.0 for 3-NFt), and one peak for pH values in between (pH 8.0–12.0 for 2-NF, pH 4.0–9.0 for 3-NFt). In the case of 1-NP, the main reductive peak is accompanied by an indistinctive second peak in the whole investigated pH range.

Based on previous CV characterization, the first, best developed peak can be ascribed to the one-step nitro group reduction to hydroxylamine (*p*_*c*_^1^, (1)), or the first step (*p*_*c*_^1*a*^, (4)) of the two-step reduction in the case of second accompanying peak (*p*_*c*_^1*b*^, (5)) in alkaline media. Only this main peak was used for analytical purposes for each tested compound due to the highest signal/noise (*S*/*N*) ratio and regular shape compared to peaks recorded at more negative potentials. The parameters of the linear regression for the dependence of the potential *E*_*p*_ of this peak on the pH of the aqueous phase are summarized in Table 2. Biphasic covering of the whole investigated pH range is notable, the intersection of the straight lines lies at about pH 7.0-8.0. This break could be caused by the change of reduction mechanism when heading to alkaline media with lack of protons as highlighted in the previous paragraph.

The optimum conditions chosen for the determination of tested compounds by DC or DP voltammetry are summarized in Table 3. The universal BR buffer was substituted by 0.05 mol L^−1^ borate buffer, pH 9.0 for the determination of 5-NQ [10] and by 0.01 mol L^−1^ NaOH, pH 12.0 for 3-NFt and 1-NP. These changes were made in order to simplify the composition of the supporting electrolyte and had no negative influence on the parameters of determination. The alkaline pH reaction of supporting electrolytes corresponds to neutral or alkaline media usually considered as optimum for the determination of nitro derivatives of aromatic compounds at mercury and amalgam electrodes (see comprehensive tables in reviews [6, 8]). Further, it can be deduced from parameters summarized in Table 2 that the difference of peak potentials for tested compounds is not sufficient for their selective determination. The optimized potentials of regeneration *E*_in_ and *E*_fin_ assure repeatability of peak heights *I*_*p*_ with relative standard deviations (RSDs) listed in Table 4. These values were evaluated from seven times repeated measurements and are <1.9% for the highest tested concentration 1 · 10^−4 ^mol L^−1^ and did not overcome 9% for the lowest measurable concentrations for all tested analytes.

Parameters of calibration dependences are summarized in Table 5, they are linear over three orders of magnitude from the concentration of ca 1 · 10^−7 ^mol L^−1^ to 1 · 10^−4^ mol L^−1^. For DPV, the linear dynamic range is always slightly wider because the regular peak-shaped signals enable evaluation of lower concentrations than the sigmoidal signals obtained by DCV. The only exception is 3-NFt, which adsorbs at the electrode surface and its linear dynamic range is therefore limited only by the concentration of 1 · 10^−5^ mol L^−1^. On the other hand, it enables its determination using adsorptive stripping differential pulse voltammetry (AdSDPV) at m-AgSAE with *L*_*Q*_ of 3 · 10^−8^ mol L^−1^ [6, 46], which is comparable to the most sensitive determinations using mercury electrodes. Possibilities of the use of adsorptive methods for the other tested analytes are under investigation, nevertheless, they succeed relatively rarely at amalgam electrodes [6, 47] even for analytes determinable by adsorptive stripping voltammetry (AdSV) at mercury electrodes, as reported for several NPAHs [10, 41, 48, 49].

**Figure 3:**
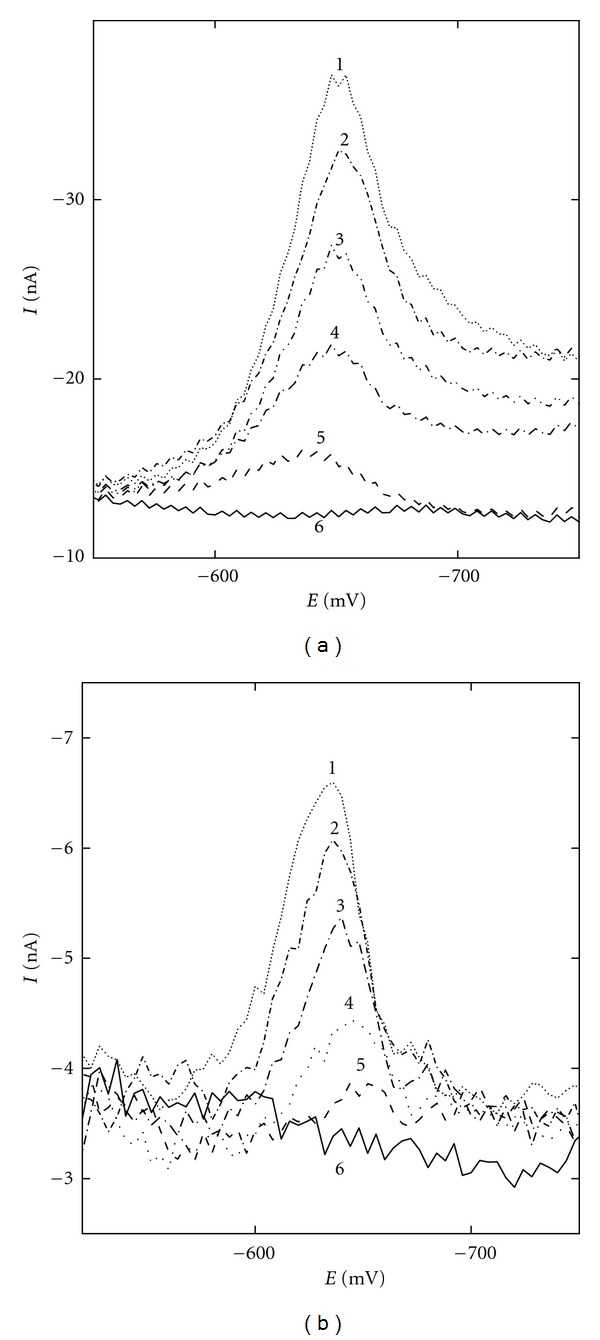
DC (a) and DP (b) voltammograms of 1-NP at m-AgSAE in a methanol—0.01 mol L^−1^ NaOH pH 12.0 (7 : 3, pH^*f*^ 12.2) mixture at the lowest attainable concentration range: (a) *c* (1-NP) = 1 · 10^−5^ (1), 8 · 10^−6^ (2), 6 · 10^−6^ (3), 4 · 10^−6^ (4), 2 · 10^−6^ (5), 0 (6) mol L^−1^, and (b) 1 · 10^−6^ (1), 8 · 10^−7^ (2), 6 · 10^−7^ (3), 4 · 10^−7^ (4), 2 · 10^−7^) (5), 0 (6) mol L^−1^. *E*_in_ = −50 mV, *E*_fin_ = −1800 mV.

The achieved *L*_*Q*_s (see Table 5) are about (2–6) · 10^−7^ mol L^−1^ for DPV and roughly one order of magnitude higher for DCV, representative voltammograms for the lowest attainable concentration range is for 1-NP and both methods depicted in Figure 3. The mentioned values of *L*_*Q*_s are consistent with *L*_*Q*_s achieved at amalgam electrodes for a number of organic analytes with reducible nitro, nitroso, or azo groups [6].

### 3.3. The HPLC-ED Method for the Determination of 1-NP, 2-NF, 3-NFt, and 5-NQ

**Figure 4:**
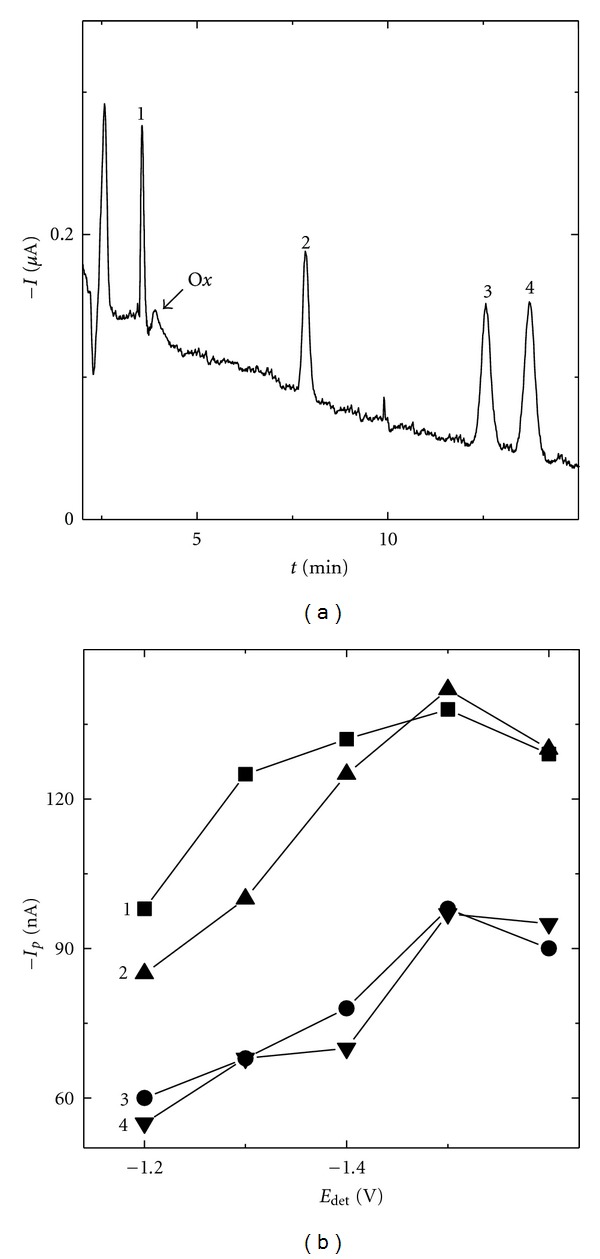
(a) Chromatogram of the mixture of 5-NQ (1), 2-NF (2), 1-NP (3), and 3-NFt (4) recorded with HPLC-ED with “wall-jet” m-AgSAE detector for concentration 5 · 10^−6 ^mol L^−1^ in drinking water after SPE: detection potential −1.5 V. *Ox*-peak of interfering oxygen. (b) Dependence of peak heights *I*_*p*_ on the detection potential *E*_det⁡_ of 5-NQ (1), 2-NF (2), 1-NP (3), and 3-NFt (4). Concentration of each analyte 1 · 10^−4 ^mol L^−1^. For (a) and (b): mobile phase 0.01 mol L^−1^ phosphate buffer pH 7.0: methanol (15 : 85; v/v), *F*_*m*_ = 1 mL min^−1^, *V*_inj_ = 20 *μ*L.

Firstly, the separation of 1-NP, 2-NF, 3-NFt, and 5-NQ was optimized using reversed C_18_ phase, which is usually used for retention of NPAHs. Problematic is the separation of 1-NP and 3-NFt, because these compounds with *M*_*r*_ of 247.26 have similarly extended aromatic system and are strongly retained at the reversed phase. Therefore, mobile phase with high content of organic phase has to be used. Baseline separation of tested analytes was achieved in fifteen minutes using 0.01 mol L^−1^ phosphate buffer, pH 7.0: methanol (15 : 85, v/v) mobile phase; the capacity factors of 5-NQ, 2-NF, 3-NFt, and 1-NP in this system are 0.84, 3.38, 6.22, and 6.96, respectively. It was verified, that the pH value of the aqueous phase has no influence on the retention times of tested analytes. The phosphate buffer, pH 7.0 was chosen with respect to the electrochemical detection, as the peak current of studied analytes measured by DCV in neutral to alkaline media is increased in comparison to the acidic ones. The chromatogram recorded with the electrochemical detector (Figure 4(a)) features another problem complicating the determination—the oxygen signal slightly coinciding with the peak of 5-NQ. The oxygen presence causing problems in HPLC-ED or FIA-ED setups was described also in other studies [11, 12] dealing with reducible analytes. Oxygen reduction current causes an increase in the background current and also limits the useful working electrode potential window. To avoid the presence of oxygen, connecting tubing in the HPLC system impermeable to oxygen has to be used and mobile phase must be degassed. Furthermore, the overflow vessel where the three-electrode system was placed was under nitrogen atmosphere. In our case, the residual oxygen is present in the injected samples (originally oxygen free), where it penetrates during the manipulation prior to the manual injection into the HPLC system.

The detection potential *E*_det⁡_ = −1.5 V was chosen based on hydrodynamic voltammograms (HDV, Figure 4(b)) of tested analytes and the highest signal-to-noise ratio. The shape of these HDVs respects the shapes of DC voltammograms as described above, that is, one signal in neutral media is recognizable. The consequent decrease of peak currents *I*_*p*_ after the height maximum at *E*_det⁡_ is reached is caused by an increase of the background current due to the cathodic decomposition (i.e., hydrogen evolution) of the mobile phase. It proceeds at relatively negative potentials due to the high content of the organic phase in mobile phase. The noise of the system is independent on *E*_det⁡_ and it amounts to ca 3.8 nA.

The repeatability of the detector response is satisfactory: For seven repeated injections of the mixture of tested analytes (*c* = 1 · 10^−4^ mol L^−1^ of each) no statistically significant change of the peak heights is observable (RSD < 3.0%) even without any m-AgSAE pretreatment.

Calibration dependences were measured under optimized conditions in the range from 1 · 10^−5^ to 1 · 10^−4^ mol L^−1^ using electrochemical detector and 1 · 10^−6^ to 1 · 10^−4 ^mol L^−1^ using UV detector, their parameters are summarized in the Table 6. Limit of quantification (10 *S*/*N*), is always 3.3 times higher than *L*_*D*_, therefore, it is not repeated in Table 6. The *L*_*D*_s achieved for electrochemical detection (from 7.8 · 10^−6 ^mol L^−1^ for 2-NF to 1.3 · 10^−5 ^mol L^−1^ for 1-NP) are higher than *L*_*D*_s achieved for UV detection (compare in Table 6) and *L*_*D*_s reported for the other common detection modes in HPLC (Table 1) [17], nevertheless, they can be lowered using preliminary separation and preconcentration of the analytes. This step is also inevitable in analysis of real matrices, because of their complex character. The samples often contain thousands of combustion products, including parent PAHs and other closely related derivatives (in particular oxygenated PAHs such as aldehydes, ketones, and carboxylic acids), which tend to coelute with NPAHs under a variety of liquid and gas chromatographic conditions and are present at concentrations 1 or 2 orders of magnitude higher than those of the nitrosubstituted compounds [13].

Thus, we tried to preconcentrate 1-NP, 2-NF, 3-NFt, and 5-NQ using solid phase extraction with endcapped C_18_ sorbent. Its amount is crucial for the recovery of extraction of 5-NQ, the most polar compound from the studied analytes. The extraction recoveries (relative standard deviations, *n* = 4) are 26.1 (15.7), 56.2 (8.1), 63.5 (13.7), 66.4 (15.1) for 5-NQ, 2-NF, 1-NP, and 3-NFt when using 100 mg of the sorbent and are raised to 84.0 (4.4), 54.4 (13.4), 79.9 (8.1), and 74.5 (8.1) using 500 mg of the sorbent and elution by 5 mL of methanol. The reduction of eluent volume to 1 mL leads to nonreproducible results. Using 500 mg of the sorbent, the calibration dependences were measured in the range from 5 · 10^−7^ to 1 · 10^−5 ^mol L^−1^ using electrochemical and spectrophotometric detection. Parameters of obtained regression dependences are presented in Table 6, an illustrative chromatogram after the SPE is recorded in Figure 4(a). Compared to standard solutions the *L*_*D*_s were lowered more than one order of magnitude for both detection modes to submicromolar concentrations and are slightly higher for ED compared to UV detection. Nevertheless, it follows from Table 1 that these detection limits can compete only with some of the voltammetric or HPLC-ED methods and could thus be applicable as an alternative in specific cases where higher content of NPAHs is expected or in screening tests on NPAHs abundance.

## 4. Conclusions 

The analysis of NPAHs in real samples requires a complex approach and can be hindered by a lack of adequate instrumental sensitivity or selectivity and limited availability of native and isotope-labeled standards. The samples often contain thousands of compounds, including parent PAHs and other closely related derivatives (in particular oxygenated PAHs such as aldehydes, ketones, and carboxylic acids). The full identification and quantification of sample composition requires often a combination of several analytical techniques. In this study, we offer an electrochemical approach using m-AgSAE as indicator electrode in batch voltammetric and flow-through HPLC-ED methods for the determination of 1-NP, 2-NF, 3-NFt, and 5-NQ, which may be used as markers of incomplete combustion. 

The *L*_*Q*_s obtained at m-AgSAE by DCV and DPV are comparable or lower than those for polarographic methods (DC tast and DP polarography offer *L*_*Q*_s in the 10^−6 ^mol L^−1^ and 10^−7 ^mol L^−1^ concentration ranges, resp.), nevertheless, they are about one order of magnitude higher than for DPV at HMDE for all studied compounds as reported in Table 1 and [8, 10, 32–34]. Attempts to lower the *L*_*Q*_s by adsorptive accumulation at the electrode surface were successful for 3-NFt [6, 46]. For further increase of sensitivity a preliminary preconcentration of studied analytes using solid phase or liquid phase extraction is recommendable, as demonstrated at HMDE for 3-NFt achieving nanomolar *L*_*Q*_s [8]. Although the selectivity of these methods is limited, they offer the possibility of inexpensive and fast screening for presence of studied analytes in environmental matrices—they can reliable prove that studied NPAHs are not present at concentrations higher than the limit of determination (which is quite frequent case in large-scale monitoring). However, if a DCV or DPV peak is found in the potential region where the signal of studied NPAH is situated, more powerful separation techniques should be used for definitive identification. 

Therefore, a HPLC method for separation of markers of incomplete combustion was developed and it was demonstrated that m-AgSAE used as indicator electrode in wall-jet amperometric detector shows good reproducibility and micromolar limits of detection for studied analytes. They can be lowered by offline preconcentration using solid phase extraction as demonstrated on model drinking water samples. Both the voltammetric and HPLC-ED methods using m-AgSAE offer notable selectivity for NPAHs, have the advantage of lower investment and running cost compared to more sophisticated spectrometric and separation techniques and, in combination with preliminary separation and preconcentration of the analytes, can represent a useful alternative for screening of NPAHs in selected real matrices. 

Our research is now focused on increasing *S*/*N* ratio and development of new designs of amperometric detectors using amalgam paste [50–52] or single crystal amalgam electrodes [53, 54]. 

## Figures and Tables

**Table 1:** Examples of limits of detection (*L*_*D*_) for 1-NP, 2-NF, 3-NFt, and 5-NQ in standards solutions by various HPLC and electrochemical methods.

Compound	Electrode or reduction technique	Detection technique	*L*_*D*_ (*μ*mol L^−1^)	References
1-NP	Pt/Rh online reducer column	HPLC-CD	0.0004	[29]
1-NP, 2-NF, 3-NFt	Pt catalyst	HPLC-FD	0.00053, 0.00047, 0.00049	[27]
1-NP	—	HPLC-TOF-MS	0.0032	[27]
1-NP, 2-NF	Offline electrochemical reduction by Ti(III) citrate	HPLC-FD	0.033, 0.032	[28]
1-NP, 2-NF	Online electrochemical reduction at porous graphite	HPLC-FD	0.0075, 0.011	[26]
1-NP, 2-NF	Glassy carbon in thin layer arrangement	HPLC-ED	0.14, 0.079	[30]
1-NP	Online electrochemical reduction at glassy carbon	HPLC-FD	0.00089	[25]
1-NP	Glassy carbon in wall-jet arrangement	HPLC-ED	0.33	[31]
1-NP, 2-NF, 3-NFt, 5-NQ	DME	DPP	0.3, 0.4, 0.1, 0.4	[10, 32–34]
1-NP, 2-NF, 3-NFt, 5-NQ	HMDE	DPV	0.06, 0.04, 0.03, 0.03	[10, 32–34]
1-NP, 2-NF, 3-NFt, 5-NQ	HMDE	AdSDPV	0.001, 0.003, 0.005, —^a^	[10, 32–34]
1-NP, 2-NF, 3-NFt	BDDFE	DPV	0.03, 0.4, 0.03^b^	[35–37]
5-NQ	m-AgSAE	FIA	3	[10]

^a^adsorption not applicable for 5-NQ; ^b^limit of quantification; CD: coulometric detection; FD: fluorescence detection; ED: electrochemical detection; TOF-MS: time-of-flight mass spectrometric detection; DME: dropping mercury electrode; DPP: differential pulse polarography; HMDE: hanging mercury drop electrode; DPV: differential pulse voltammetry; AdSDPV: adsorptive stripping DPV; BDDFE: boron-doped diamond film electrode; m-AgSAE: mercury meniscus modified silver solid amalgam electrode; FIA: flow injection analysis.

**Table 2:** The parameters of the linear regression *E*_*p*_  [*mV*] = *a*  pH + *b* for the dependence of peak potential *E*_*p*_ of the first main nitro group reduction on the pH of the aqueous phase of the supporting electrolyte. Supporting electrolyte: BR buffer-methanol in ratios 1 : 1, 3 : 7, and 1 : 9 for 2-NF, 1-NP, and 3-NFt, respectively.

Analyte	pH of the aqueous phase	Slope (*a*) (mV^−1^)	Intercept (*b*) (mV)	Correlation coefficient
1-NP	2.0–7.0	−62.3	−80.1	−0.9950
	8.0–12.0	−40.9	−268.3	−0.9915
2-NF	1.0–7.0	−66.9	−102.0	−0.9981
	8.0–12.0	−32	−272.6	−0.9991
3-NFt	2.0–8.0	−50.1	−227.5	−0.9961
	9.0–11.0	−32	−326.0	−0.9881

**Table 3:** Regeneration potentials, optimum supporting electrolyte and potential of the main reductive peak of nitro group for the DC voltammetric and DP voltammetric determination of 1-NP, 2-NF, 3-NFt, and 5-NQ at m-AgSAE.

Analyte	Regeneration potentials (mV)		Optimum supporting electrolyte	Peak potential (mV)^a^
	*E*_in_	*E*_fin_		
1-NP	−50	−1800	Methanol—0.01 mol L^−1^ NaOH pH 12.0 (7 : 3)	−650
2-NF	−200	−1500	Methanol—BR buffer pH 10.0 (1 : 1)	−680
3-NFt	−350	−1600	Methanol—0.01 mol L^−1^ NaOH pH 12.0 (9 : 1)	−660
5-NQ^b^	0	−1350	0.05 mol L^−1^ borate buffer pH 9.0	−605

^a^For DCV and concentration 1 · 10^−4^ mol L^−1^; ^b^from [10].

**Table 4:** Repeatability (*n* = 7, relative standard deviation, RSD) of peak height *I*_*p*_ for DC voltammetric and DP voltammetric determination of 1-NP, 2-NF, 3-NFt, and 5-NQ at m-AgSAE. Conditions for each analyte are listed in Table 3.

Analyte	DCV		DPV	
	RSD of *I*_*p*_ for *c* = 1 · 10^−4^ mol L^−1^	RSD of *I*_*p*_ for (*L*_*Q*_-close *c* in mol L^−1^)	RSD of *I*_*p*_ for *c* = 1 · 10^−4^ mol L^−1^	RSD of *I*_*p*_ for (*L*_*Q*_-close *c* in mol L^−1^)
1-NP	1.74	7.89 (8 · 10^−7^)	1.52	9.12 (1 · 10^−7^)
2-NF	1.32	6.88 (1 · 10^−6^)	0.96	3.15 (1 · 10^−7^)
3-NFt	1.55	8.14 (4 · 10^−7^)	1.10	7.16 (4 · 10^−7^)
5-NQ^a^	1.83	9.31 (4 · 10^−7^)	1.98	8.89 (2 · 10^−7^)

^a^ from [10].

**Table 5:** *L*_*Q*_ for the DC voltammetric and DP voltammetric determination of 1-NP, 2-NF, 3-NFt, and 5-NQ at m-AgSAE. Conditions for each analyte are listed in Table 3.

Method	Analyte	LDR^a^ (*μ*mol L^−1^)	Slope (mA mol^−1^ L)	Intercept (nA)	Correlation coefficient	*L*_*Q*_ (*μ*mol L^−1^)
DCV	1-NP	1–100	2.50	9.9	0.9972	3
	2-NF	1–100	1.11	2.8	0.9969	2
	3-NFt	0.4–10	2.30	2.9	0.9933	0.4
	5-NQ^b^	0.6–100	1.33	6.8	0.9919	0.5

DPV	1-NP	0.1–100	2.65	7.8	0.9911	0.6
	2-NF	0.1–100	1.20	1.4	0.9976	0.2
	3-NFt	0.4–10	5.50	5.7	0.9985	0.4
	5-NQ^b^	0.2–100	1.38	–2.5	0.9925	0.3

^a^Linear dynamic range, ^b^from [10].

**Table 6:** Parameters of calibrations dependences for the determination of 5-NQ, 2-NF, 1-NP, and 3-NFt using HPLC-ED with “wall-jet” m-AgSAE detector (A) under optimized conditions in deionized water as an ideal matrix and (B) for determination in drinking water after SPE. Mobile phase 0.01 mol L^−1^ phosphate buffer pH 7.0: methanol (15 : 85; v/v), *F*_*m*_ = 1 mL min^−1^, *V*_inj_ = 20 *μ*L, detection potential −1.5 V, responses evaluated from the peak heights.

	Analyte	Linear dynamic range (*μ*mol L^−1^)	Slope^a^	Intercept^b^	Correlation coefficient	*L*_*D*_ (*μ*mol L^−1^)
(A) Optimized conditions in deionized water						

Electrochemical detection	5-NQ	10–75	–1.28	–0.6	–0.9972	8.9
	2-NF	25–100	–1.45	–2.1	–0.9798	7.8
	1-NP	25–100	–0.91	–15.5	–0.9935	12.5
	3-NFt	25–100	–1.00	1.0	–0.9985	11.4

UV detection	5-NQ	1–100	0.502	–0.12	0.9999	1.6
	2-NF	1–100	0.423	–0.19	0.9998	1.9
	1-NP	1–100	0.882	–0.39	0.9999	0.9
	3-NFt	1–100	0.943	–0.12	0.9999	0.8

(B) Determination in drinking water after SPE						

Electrochemical detection	5-NQ	0.5–5	–25.9	2.3	–0.9997	0.66
	2-NF	2–10	–20.7	–13.0	–0.9957	0.83
	1-NP	2–10	–20.3	–15.2	–0.9823	0.84
	3-NFt	2–10	–16.0	7.8	–0.9766	1.1

UV detection	5-NQ	0.5–10	10.2	–4.0	0.9829	0.38
	2-NF	0.5–10	8.0	–4.2	0.9937	0.50
	1-NP	0.5–10	16.0	–3.1	0.9940	0.24
	3-NFt	0.5–10	21.2	–5.0	0.9882	0.18

^a^ For electrochemical detection in nA *μ*mol^−1^ L and for spectrophotometric detection in mA.U. *μ*mol^−1^ L.

^b^ For electrochemical detection in nA and for spectrophotometric detection in mA.U.
